# Care as a Central Concept: Dimensions, Inequalities and Challenges in Chronic Care in Contemporary Societies: A Narrative Review

**DOI:** 10.3390/healthcare14030359

**Published:** 2026-01-30

**Authors:** Dolores Torres-Enamorado, Rosa Casado-Mejía

**Affiliations:** Nursing Department, Faculty of Nursing, Physiotherapy, and Podiatry, University of Seville, 41009 Seville, Spain; rcasado@us.es

**Keywords:** informal care, chronic care, feminist economics, gender inequalities, health policy

## Abstract

**Highlights:**

**What are the main findings?**

- Care is a structural, political, and transnational category that sustains life and healthcare systems.
- A transformative analysis of care must address its material dimension (as work), its subjective dimension (including emotional bonds and moral responsibility), and its political dimension (shaped by power relations).

**What are the implications of the main findings?**

- Advancing towards policies of social co-responsibility is essential, not only by promoting caring masculinities but also by strengthening public services, professionalizing the care sector, and formally recognizing care work.
- Further research is needed to promote the recognition, redistribution, and socialization of care, which are essential for social justice and for safeguarding the dignity of both caregivers (predominantly women) and care recipients.

**Abstract:**

**Background/Objective**: Feminist theories and feminist economics have contributed to making visible the structural relevance of care work in sustaining capitalist societies and social reproduction, arguing that care must be addressed as a political phenomenon rather than a merely domestic issue. This perspective is particularly pertinent in contemporary healthcare, where chronic care represents one of the major public health challenges in a context of population ageing and increasing prevalence of chronic diseases. The aim is to contribute to a critical understanding that can support the development of public policies recognizing care as a fundamental pillar of socio-healthcare provision and as a matter of collective responsibility. **Methods**: A narrative literature review with a critical feminist approach was conducted using PubMed/MEDLINE, Scopus, and Web of Science. **Results**: A total of 299 records were identified, of which 30 studies were included following screening and eligibility assessment. Care is an essential element for sustaining life, although it has historically been rendered invisible, feminized, and relegated to the private sphere. Chronicity requires simultaneous consideration of the material dimension of care (as work), the subjective dimension (including emotional bonds and moral responsibility), and the political dimension (shaped by power relations). Global care chains reveal persistent inequalities related to gender, class, and race. **Conclusions**: Care is a structural, political, and transnational category that sustains life and healthcare systems. In the field of chronic care, the recognition, redistribution, and socialization of care are essential for achieving social justice and for safeguarding the dignity of both caregivers—predominantly women—and care recipients.

## 1. Introduction

Currently, one of the main global problems—or challenges—in public health is represented by chronic care needs, derived primarily, though not exclusively, from population ageing. The World Health Organization estimates that by 2030 one sixth of the world’s population will be aged 60 years or older, and that this figure will double by 2050 [1].

This demographic ageing entails social, economic, and political changes, as increased life expectancy does not always go hand in hand with good quality of life, leading to a greater prevalence of chronic diseases and their potential consequences in terms of acquired dependency [2].

The growing number of older people with dependency creates a need for caregivers to meet the assistance requirements of dependent individuals. Care is an essential economic activity in all societies, and paradoxically, those who need it most are often the least able to obtain it through the market. Consequently, the foundation of dependency care is located within the family, particularly among women, who—without receiving any form of remuneration—are responsible for meeting the basic and instrumental needs that dependent individuals are unable to perform independently [3].

In recent decades, feminist movements and feminist economics have contributed to making visible the structural relevance of care work in sustaining capitalist societies and social reproduction, highlighting the need to address care as a political phenomenon rather than a merely domestic one [4,5,6]. This reflection is particularly relevant in contemporary healthcare, where chronic care represents one of the major public health challenges in the context of demographic ageing and increasing prevalence of chronic diseases [3].

This article examines family care from three key perspectives: its central and political nature, its core dimensions (material–physical, subjective–relational, and political), and one of the social responses that has led to the emergence of global care chains that reconfigure inequalities at a transnational scale [7,8,9,10].

From these perspectives, the following questions arise: is the care of people with chronic conditions an individual or family issue, or a public health concern? Are there sufficient public policies to address social care needs? Does the fact that most caregivers are women influence the inadequacy of public policies? What dimensions and scope does care encompass? The aim is to contribute to a critical understanding that can support the development of public policies recognizing care as a fundamental pillar of socio-healthcare provision and as a matter of collective responsibility.

## 2. Materials and Methods

### 2.1. Study Design

A narrative literature review with a critical and feminist approach was conducted, aimed at analysing care as a central and political concept within the context of chronic care. This methodological design allows for the integration of theoretical, empirical, and normative evidence from different disciplines—public health, nursing, sociology, feminist economics, and gender studies—in order to provide a broad and contextualised understanding of care and the structural inequalities that shape it [11,12].

Narrative reviews are particularly appropriate when the objective of the study is not the quantitative synthesis of results, but rather the critical interpretation of conceptual frameworks, the identification of power relations, and the generation of theoretical reflection—key aspects for the analysis of care, chronicity, and global care chains [4,5].

The feminist approach was incorporated as an epistemological framework to make visible power relations, gender inequalities, and processes of invisibilities that permeate the social organization of care, particularly in contexts of chronicity and prolonged dependency [13]. Seminal works in feminist economics, the sociology of care, and care ethics [4,5,8,9,10,14,15,16,17] were used to predefine the categories for the classification and analysis of the selected articles.

### 2.2. Search Strategy

The literature search was conducted between September and October 2025 in the databases PubMed/MEDLINE, Scopus and Web of Science, selected for their relevance in the fields of health and social sciences.

Specifically, searches in Web of Science and Scopus were conducted on 29 September 2025, while the search in PubMed/MEDLINE was carried out on 6 October 2025.

**Figure 1:**
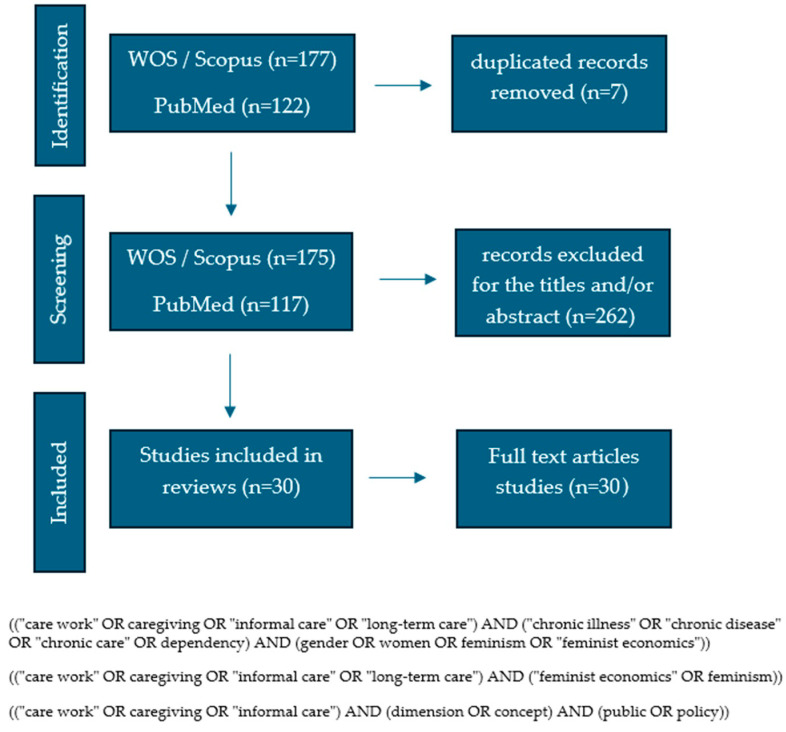
Flowchart of the document selection process.

Combinations of controlled vocabulary terms (MeSH) and keywords in Spanish and English were used, including: care, care work, informal caregiving, chronic care, long-term care, gender, feminist economics, global care chains, migration, care policies, and health inequalities. Terms were combined using Boolean operators (AND, OR) to maximise search sensitivity and obtain the search string (View Figure 1). This strategy enabled the retrieval of relevant literature from both the healthcare field and social and gender studies.

### 2.3. Inclusion and Exclusion Criteria

Inclusion criteria:

- Peer-reviewed scientific articles.
- Literature reviews
- Qualitative, quantitative, and theoretical studies addressing chronic care from a social, gender, or political perspective.

Exclusion criteria:

- Studies focused exclusively on clinical interventions without analysis of the social context or family care work.
- Publications without access to the full text.
- Duplicate documents or sources containing redundant information.
- Studies not related to chronic care or to the structural analysis of care.
- No language restrictions apply.

No strict time limit was established; instead, priority was given to the inclusion of foundational classical works alongside recent relevant literature in order to capture the conceptual evolution of care as an analytical category.

### 2.4. Selection and Data Analysis Process

Although this study follows a narrative review design, the selection process and thematic synthesis were conducted in a systematic manner in order to enhance transparency and analytical rigor.

The selection of documents was carried out in two phases: an initial screening of titles and abstracts, followed by an in-depth review of the full texts that met the inclusion criteria. The analysis was conducted through a critical and thematic reading, identifying analytical categories defined a priori based on the feminist theoretical framework:

- Care as central and political concept.
- Dimensions of care (material–physical, subjective–relational, and political),
- Chronic care,
- Global care chains and intersectional inequalities

These categories were continuously refined throughout the analytical process, allowing for the integration of contributions from different theoretical approaches and empirical evidence. In addition, to contextualize the current state of the issue and inform the subsequent discussion, key works in feminist economics, sociology of care, and care ethics were consulted. Institutional repositories and grey literature sources (reports from international and national organizations related to dependency, chronicity, and care policies) were also examined, due to their importance in the analysis of public care policies.

The adopted approach was interpretative and reflexive, aimed at identifying patterns, tensions, and convergences across the different bodies of literature [7,18].

### 2.5. Ethical Approach and Scientific Rigor

As this study was based on secondary data sources, approval from a research ethics committee was not required. Nevertheless, the principles of academic integrity, transparency, and accurate citation of sources were strictly observed.

The rigor of the analysis was ensured through source triangulation—including scientific articles, theoretical literature, and institutional reports—and by selecting widely recognized reference works in the fields of care studies and public health [19].

## 3. Results

The search process yielded a total of 299 records. After screening titles and abstracts and removing 7 duplicates, the eligibility of 30 full-text articles was assessed, which were included in the final synthesis (Figure 1).

Table 1 identifies the selected articles by their authors and year, describing: “Study Design/Approach”, “Population/Focus”, “Dimension of Care Addressed”, “Key Contributions” and “Relevance for chronic care with feminist perspective”.

### 3.1. Care as a Central and Political Concept

#### 3.1.1. Care as a Structural Category for Sustaining Life

Care constitutes an essential element for the sustainability of life and is indispensable for both individual and collective well-being. However, its importance has historically been rendered invisible, feminized, and relegated to the private sphere [20]. Feminist economics has highlighted that this invisibilization responds to androcentric logics that prioritize market production over social reproduction, thereby marginalizing essential activities such as everyday care, domestic work, and emotional support [21,22].

In the field of chronic care, this invisibilization implies that a substantial proportion of the tasks required for managing chronic illnesses fall upon family members—particularly women—who, despite being essential for maintaining health and quality of life, receive neither recognition nor compensation [23,24,25,26].

#### 3.1.2. Care as a Public Rather than a Private Issue

Addressing care as a political issue implies recognizing that its organization cannot rely exclusively on the private sphere. Even though women have internalized caregiving as a gender mandate [27], the sustainability of chronic care requires continuous attention, complex management, and constant presence. This highlights the need for public policies that guarantee adequate services, support for caregivers, and social and gender co-responsibility. It is not a private matter, it is a public health issue [28,29,30].

The persistence of familistic models places the responsibility for care almost exclusively on women within families, generating inequality, overload, and limitations on their autonomy [31]. This situation is particularly critical in contexts of chronicity, where care may extend over years or even decades [32]. Caring causes multiple negative consequences for caregivers across physical, economic, emotional, and social domains [28,33,34].

**Table 1:** Characteristics of Included Studies.

Author(s), Year	Country/Region	Study Design/Approach	Population/Focus	Dimension of Care Addressed	Key Contributions	Relevance for Chronic Care with Feminist Perspective
[35]	New Zealand	Qualitative feminist intersectional research	Formal and informal care	Relational	The study shows that caregiving poses significant risks to caregivers’ health and wellbeing and may exacerbate existing gendered and racial health inequities. These impacts are shaped by socio-cultural pressures and intersecting systems of power, highlighting the need for greater institutional support and resources, particularly for caregivers with double care responsibilities.	Provides empirical evidence on how double-duty caregiving intensifies emotional, physical and economic burdens for women, particularly Māori and migrant caregivers, highlighting care as a relational practice shaped by gendered, cultural and structural inequalities and positioning caregiver support as a social justice and health promotion issue.
[36]	China	Concept mapping study	Carers, patients, professionals and community members	Political	The study highlights the potential of social capital to support informal caregivers of people with advanced chronic illnesses. While caregiving burden is well documented, the findings underscore the need to better identify and mobilize specific dimensions of social capital that can effectively address caregivers’ health and support needs at the community level.	The different dimensions of social capital can serve as a framework for developing social care programs and policies to support informal carers.
[37]	Australia	Qualitative research	Female migrant workers	Political	The article examines the lived experiences of Asian female aged care workers through the concept of carescape and a theory of agency. It shows how social and institutional structures both constrain and enable workers’ agency, shaping access to the aged care workforce and the development of social support networks and resilience.	Institutional and social structures are related to the agency and resilience of female migrant workers.
[38]	South Africa	Qualitative research	Caregivers and older care recipients	Relational and political	The study shows that conceptualizations of good care are shaped by, yet also challenge, prevailing policy and cultural ideals. It emphasizes the need for care policies and interventions that address the broader care ecology, particularly the formal carescape, and advocates relational approaches that balance the needs, rights, and experiential knowledge of both caregivers and care recipients.	Based on the theory of the ethics of care and within a framework of the ecology of care, the need for policies focused on caregivers and care recipients is highlighted.
[39]	Greece	Quantitative observational research, cross-sectional type	Family caregivers of cancer patients	Relational	Burden increase was found to be related to stress increase. Younger participants and patients’ female spouses had higher levels of stress. The present research also found that stress related to financial issues had an important role. Family caregiver support is found to be a matter of great importance, and healthcare professionals have to pay attention to their needs.	Provides empirical evidence that high levels of stress and caregiving burden among informal cancer caregivers are strongly associated with poorer quality of life, highlighting the emotional and material demands of chronic and highly complex home-based care and the need for greater caregiver support.
[40]	USA	Essay	Home care	Political	The article argues for a normative shift from individualistic to systemic approaches to care work, emphasizing the social, economic, and historical forces shaping contemporary care. It contends that improving working conditions through this lens can reduce tensions between caregivers and care recipients and better support feminist ethical ideals of care.	Re-centers home care within bioethics by framing care as socially necessary work shaped by political economy, gendered and racialized inequalities, and labor conditions, arguing for systemic rather than individualistic approaches to improving care in aging and chronic illness contexts.
[33]	Slovakia	Quantitative observational research, cross-sectional type	Informal caregivers—older adults with dementia	Relational, material and political	Implementing psycho-social and educational public health interventions focused on strengthening social support and maintaining positive perceptions of caregiving can help reduce the increased risk of caregiver burden in informal caregivers of older adults with dementia.	Provides empirical evidence that perceived social support and positive caregiving experiences significantly reduce caregiver burden in informal caregivers of people with dementia, highlighting the relational and emotional dimensions of chronic care and the structural reliance on informal caregiving.
[41]	International (Europe & Latin America)	Integrative Literature Review	Informal care	Political	The articles highlight the potential of personal assistance while stressing the need for greater resources to avoid its restriction to high-income contexts. From a feminist disability care ethics perspective, they show that informal care is largely provided by racialized migrant women and argue for a shift from individual and family responsibility toward collective models of care grounded in interdependence and autonomy.	Identifies politico-epistemic tensions between personal assistance and care from feminist and disability perspectives, highlighting care as a collective, interdependent and structurally unequal practice that requires public responsibility and policy-level transformation.
[42]	USA	Theoretical-analytical essay	Family caregiving	Political	The study analyzes older adult care work in the United States as a multilayered system of exploitation that reinforces gendered and racialized hierarchies under capitalist expansion. It contrasts two possible trajectories: increased commodification of care under neoliberalism versus the development of social welfare policies that reduce women’s reproductive labor burden and challenge existing inequalities.	Conceptualizes older adult care as an instrument of capital accumulation under neoliberal capitalism, demonstrating how unpaid and paid care work is structurally gendered, racialized and exploited, and framing care as a central political and structural issue in aging and chronic care systems.
[24]	Mexico	Qualitative research	Informal caregivers	Material-physical/relational	The paper examines how women’s paid and unpaid work trajectories are intertwined over the life course in Mexico, highlighting intergenerational differences and social inequalities. From a feminist economics perspective, it demonstrates the central role of domestic and unpaid care work in sustaining life and its relevance for economic analysis.	Shows material demands of chronic caregiving
[43]	Chile	Qualitative research	Informal caregivers	Political	The study identifies a complex web of daily care activities that generates significant overload for informal caregivers, with personal, family, and economic consequences. It shows that the sexist social organization of care produces gendered and precarious caregiving roles, framing informal care for dependent adults as a structural and political issue rather than a personal one.	The article raises, from a feminist perspective, how the growing crisis of care, accentuated in neoliberal states, generates an absorption of this problem by women, especially when it comes to dependent adults.
[44]	China	Bioethical analysis	Family care	Political	This article identified the constraint of gender hierarchy and its intersection with external social structure that exacerbate gendered oppression and exploitation of female labour in rural China. Normatively, it argues that the current configuration of rural family care, featured by structural impediments and exploration of female labour, is unjust. Some policy recommendations are proposed to empower caregivers and advance care for rural older people.	Relevant to ethical challenges in chronic illness care. This article adopted an empirical ethical approach that integrates ethnographical investigation and feminist ethical inquiry.
[25]	Internacional	Essay	COVID-19 Care	Political	The study highlights how the COVID-19 pandemic intensified existing structural inequalities and exposed the central role of paid and unpaid care work in sustaining societies and economies. From a feminist economics perspective, it underscores the need for policy approaches that prioritize social reproduction and human wellbeing over market-centered production.	It underscores the need for a structural reorganization of the economy that places care at its core.
[22]	Canada	Qualitative research	Family Caregivers	Political	They identify three themes central to a relational ethic of home support on two rural islands: the strength of intergenerational connections, community-embedded relationships, and care as compassionate civic engagement. Within each theme, they consider how shifting policy structures inform changes over time in the nature and delivery of home support.	Highlights emotional burden in long-term and chronic care
[31]	Europe	Review	Informal caregivers	Political	The study shows that demographic and socio-cultural changes are increasing dependency while threatening the availability of informal caregivers, with care responsibilities falling predominantly on women. From a gender perspective, it highlights how caregiver burden reinforces global gender inequalities and emphasizes the need for public, community, and health policy interventions—particularly within nursing practice—to promote more shared and equitable care arrangements.	It is advocated to include a gender perspective in the development of intervention plans to improve the quality of life of caregivers.
[30]	Europe: Austria, Germany, Italy (receiving countries), Poland, Romania (sending countries)	Scoping review	Migrant caregivers, care chains	Political	The study indicates that shortages of care workers, combined with cash benefits and strong norms of family responsibility, contribute to increased reliance on migrant carers, often in low-skilled or informal positions. It highlights how the COVID-19 pandemic exposed the fragility of these labor arrangements and underscores the need for policies that integrate migrant long-term care workers into public health research and European health workforce governance.	Demonstrates the structural dependence of European long-term care systems on migrant carers and introduces a trans-sectoral governance framework that links care systems, labour markets and migration policies, highlighting care as a central political and public health issue.
[32]	Netherlands	Review	Informal caregivers	Physical/relational	The study identifies caregiving duration and patients’ levels of dependency as key predictors of caregiver burden, with behavioral and cognitive factors shaping care demands. It emphasizes the need for disease-specific support interventions and highlights the potential of changing gendered role expectations—particularly increasing men’s involvement—to reduce the disproportionate burden borne by women.	Predictors of caregiver burden are identified.
[45]	China	Qualitative research	Live-in migrant care workers	Relational	Emotional labor allowed live-in migrant care workers to avoid conflict with older people, and to further protect their own welfare and that of others. There is a need to develop culturally appropriate interventions to empower live-in MCWs to deliver emotional labor in a moral manner.	Highlights the significance of empowering live-in migrant care workers by training them in ways that will help them to adapt to working conditions where they will encounter diverse customs and older people who will develop an increasing dependence on them. Analysis drawing on feminist phenomenology
[46]	Spain	Review	Care concept	Relational	They propose to extend the concepts of analysis from household production to any form of social interaction. So they define these concepts by the following categories of analysis: (a) the centrality of people’s needs in action; (b) the centrality of target achievement of the action; (c) the centrality of universal standards in action, (d) the centrality of the objects in dispute in the action.	In this paper they construct the concepts of care, provision and service as tools for analysis of social interaction from a gender perspective
[47]	Spain	Qualitative research	Informal caregivers	Relational	Results indicated that women report more guilt than men for abandoning family and friends, and because of their relationship with the dependent person. However, with respect to nursing home placement, no difference was observed as a function of gender. The high incidence of caregiver guilt needs to be addressed by health professionals to avoid the emergence of other mental health issues.	This study analyzes guilt among family caregivers of dependent patients, from a gender perspective
[48]	USA	Quantitative epidemiological observational research.	Informal caregivers	Political	The study shows that population ageing is increasing demand for informal family care while the pool of caregivers is shrinking, with women reporting higher and relationship-dependent levels of burden, particularly in mental and neurodegenerative conditions. It adopts a structural and global perspective, highlighting how welfare systems outsource care to families—especially in low- and lower-middle-income countries—and conceptualizes informal care as an uncompensated social asset sustaining healthcare systems amid ageing and chronic illness.	Provides robust cross-national evidence on the objective and subjective burden of informal caregiving in chronic illness, demonstrating strong gendered and socioeconomic inequalities and highlighting the structural dependence of health systems on unpaid family care.
[49]	Spain	Qualitative research	Migrant caregivers	Relational- Political.	The most important factors for carers’ health were the migration process and care tasks. Interpersonal relationships constituted the principal factor affecting the health of all involved: Good and egalitarian interpersonal relationships are a protective factor for health.	Seeking to understand the effects of care within the family provided by live-in female immigrants on elderly dependents and their families and the carers themselves, from a gender perspective.
[26]	UK	Theoretical	Theories of recognition. Dignity and care	Relational	The article shows that postmodern feminist and recognition theories challenge dominant liberal approaches by foregrounding the contingent, affective, and reflexive dimensions of care work. It reconceptualizes dignity as a contested and politicized concept, shifting debates on care from normative injunctions toward critical analyses of morally complex practices shaped by power relations.	Reframes dignity as a relational and politicised practice of care shaped by recognition, power and organisational conditions. The need to review the debate on dignity through the lens of feminism and theories of recognition is argued.
[34]	UK and Netherlands	Qualitative research. Binational comparative study.	Caregivers	Political	The study compares caregiver policies and experiences in England and the Netherlands, showing how European welfare states struggle to reconcile increased labor market participation with growing demands for family care. Drawing on the concepts of doulia and doulia rights, it finds that while both policy models have distinct strengths and weaknesses, neither demonstrates a strong commitment to protecting caregivers’ rights to provide care without economic hardship.	Applies the concept of “doulia rights” to empirical evidence, demonstrating that welfare and labour policies in different European regimes fail to adequately support informal caregivers combining work and chronic care, thereby revealing the structural dependence of care systems on unpaid caregiving.
[28]	USA	Conceptual and narrative public health analysis	Care model	Political	Health systems are structurally dependent on informal care. Caregiving is an emerging public health issue involving complex and fluctuating roles. Caregiving must be considered in the context of life span needs that vary according to the ages, developmental levels, mental health needs, and physical health demands of both caregivers and care recipients.	Proposal of a triadic care model (family caregiver—care recipient—professionals), integrated within a framework of social, political and demographic forces.
[27]	USA	Qualitative research	Female family caregivers	Political	Gender identity is a powerful aspect of self that shapes values, attitudes, and conduct. Family caregivers, particularly women, tend to forgo institutionalization of care recipients even when care demands are overwhelming. The reluctance of women to relinquish care raises questions about the relationship between gender identity and the bearing of burden.	Highlights that caregivers had internalized stereotypical female gender traits that support and facilitate the enduring of burden.
[29]	Spain	Theoretical–empirical public health study, using a gender and social class perspective	Care concept and caregivers	Political	Informal health care poses two key questions with regard to the issue of equity: differences in the burdens borne by men and women, which contribute to gender inequality and, depending on their educational and socio-economic level, inequities in their ability to choose and gain access to needed resources and support services, thus contributing to social class inequalities. Distributing the burden of caregiving between men and women, and between the family and the state, constitutes a crucial debate in public health.	Conceptualizes informal care as an invisible health care system and provides empirical evidence that caregiving in chronic and long-term care contexts is structurally feminized and socially stratified, generating profound gender and class inequalities and calling for policy-level redistribution of care responsibilities.
[21]	USA	Essay/Theoretical analysis	People with disabilities and caregivers. Ethics of care	Political	Persons with disabilities and those who help care for them must form an alliance to advance their common interests. This alliance can gain insight and inspiration from feminist thought insofar as caretaking is literally linked to problems of the representation of caretaking as “women’s work,’’ and more philosophically, by borrowing from the toolbox of feminist social, political, and economic analyses.	Provides normative basis for care policies in chronic care. Develops ethical framework linking care and justice.
[23]	USA	Feminist theory	Care ethics	Political—structural	Feminist critique of care ethics traditions	Frames care as political issue beyond private sphere
[20]	USA	Review	Family caregivers	Material-physical	The review highlights the central role of family caregivers—predominantly women—in sustaining long-term care for chronically ill elders and children. While caregiver burden is well documented, it underscores the limited attention given to intervention strategies and emphasizes the need for nursing practices and research focused on reducing caregiver burden.	Connects caregiving workload with chronic disease

### 3.2. Dimensions of Care: Contributions to Understanding Chronic Care

#### 3.2.1. Material–Physical Dimension

From this perspective, care is understood as concrete work that consumes time, energy, and material resources [29]. Care as work involves specific and routine actions such as personal hygiene, feeding, mobilization, environmental cleaning, and logistical management [22]. In chronic care, this dimension is intensified due to the prolonged duration and progressive complexity of care needs [32].

Caregivers face repetitive, physically demanding, and highly intensive tasks. Partial outsourcing of these activities through services such as home care or professional support is often insufficient or inaccessible and may even increase the family burden. In many cases, outsourcing is not feasible due to economic constraints [28,29,30,31,32,33,34], and gender-based prioritization of self-care [39]. For example, male caregivers tend to externalize substitute care for basic activities and promote self-care, even when this results in economic impoverishment. Greater participation of men should be encouraged, without prejudice to other public accountability strategies [29,30,31,32].

Provision relationships are often instrumental in nature, aimed at achieving outcomes without adequately considering their impact on individuals and the surrounding environment [43]. All human activities should be considered through relationships of care, service, and provision [46]. Policies promoting personal autonomy and addressing dependency situations should take into account both care recipients and caregivers [38]. The meeting point between both perspectives is interdependence and autonomy; on the one side, for people with disabilities, and on the other, for the women profiled as the main caregivers [21,41]. Policies should be adaptable to specific needs and contexts rather than based on generalized or abstract standards. Furthermore, they must consider their intersectional nature [35,36,42].

#### 3.2.2. Subjective–Relational Dimension

Care for dependent older adults is often a prolonged process that becomes embedded in caregivers’ daily routines, turning into the central axis around which all other daily activities revolve [34].

Care involves emotional bonds, moral responsibility, and, in many cases, a gender mandate prescribing that women “must” provide care. They internalize stereotypical female gender traits that support and facilitate the assumption of the burden [27]. This mandate gives rise to feelings of guilt, self-demand, and emotional exhaustion [47].

Chronic care requires prolonged emotional presence, management uncertainty, and providing companionship in the face of often intensified illness and progressive deterioration. This emotional dimension is a source of satisfaction for the caregiver, but also of vulnerability. While this emotional dimension can be a source of satisfaction, it also constitutes a significant source of vulnerability [29]. Family caregivers of individuals with conditions such as dementia, cancer, or neurological diseases face increased risks of anxiety, depression, compassion fatigue, and social isolation. The chronic nature of care transforms it into a persistent stressor, generating physical and emotional burden that affects all spheres of caregivers’ lives [43,47].

The subjective component of family care is increasingly recognized as a key determinant of caregiver burden [35]. Therefore, considering subjectivity within the analysis of chronic care is becoming an increasingly relevant issue. Accordingly, caregiving itself does not inherently produce observed morbidity; rather, it is the conditions under which care is provided that generate overload [20].

#### 3.2.3. Political Dimension

Care is shaped by power relations: who provides care, who receives it, who coordinates it, who makes decisions, and who recognizes or remunerates it. This sets up a gender hierarchy, which intersects and interrelates with the external social structure [26,44]. Certain public policies contribute to the crystallization of gender roles by relegating women to care work and the domestic sphere, revealing that labor market logic largely responds to a male breadwinner model without family responsibilities [29]. The lack of recognition and feminization of care generate and reproduce forms of exploitation and inequality [25,42]. In chronic care, this dimension becomes particularly evident through the precarization of caregivers and the insufficiency of institutional support [34,48]. It is necessary to replace the dominant individualistic thinking with systemic thinking [38,40,41,43].

Gender, and its intersections with other critical identities such as race, ethnicity, and nationality, are the basis of exploitation and marginalization in modern capitalist systems. It should move towards more comprehensive social welfare policies that alleviate women’s reproductive labor burden and begin to dismantle gender and racialized hierarchies [25,42].

### 3.3. Global Care Chains and Their Impact on Chronic Care

The growing demand for care in ageing societies has driven the migration of women to work as caregivers, giving rise to global care chains. Migrant populations have become a fundamental pillar of the current chronic care system. Predominantly women, migrant caregivers take on a large part of the home care work under precarious labor conditions, characterized by long working hours and limited social protection [30,49].

Global care chains reveal inequalities related to gender, class, and race: migrant women occupy highly feminized and precarious care positions, while families with fewer resources assume a greater direct care burden [49]. Ultimately, chronic care is sustained at the cost of structural inequalities [42].

The provision of chronic care by migrant workers highlights the insufficiency of public support services and the need to rethink the organization of care within health systems [38]. Their indispensable role in sustaining care networks calls for greater institutional recognition and the implementation of policies ensuring labor rights, administrative regularization, and specific training [30], that facilitate the agency, adaptation and transformation of these migrant women caregivers [37].

## 4. Discussion

Feminist theories agree that care, despite being essential for the sustainability of life and for individual and collective well-being, has been undervalued and rendered invisible because it is primarily performed by women. As a result, care has been relegated to the private sphere. Prioritizing market production over social reproduction, thereby marginalizing essential activities such as care, reflects androcentric logics, as shown by our results and in line with well-established contributions from feminist economists [4,5,14]. Moreover, indexed articles addressing family care from a feminist economics perspective remain scarce, reinforcing the view that this area of analysis has not yet been sufficiently developed.

The epidemiological transition toward chronic diseases requires models of continuous care, integrated health and social care coordination, and support for families [50]. However, public systems do not adequately meet this demand, which exacerbates caregiver burden and leads to the emergence and consolidation of new forms of inequality.

Problematizing the phenomenon of care and reflecting on its challenges [51], while integrating a feminist and intersectional perspective into chronic care, makes it possible to identify how inequalities are produced and how they could be transformed through redistributive policies and models of social co-responsibility [14,52,53,54]. Advancing toward the democratization of care implies recognizing the social centrality of care and the socialization of responsibility for caregiving [55], As Joan Tronto argues [56], democracy cannot be understood without care, going beyond a more equitable individual distribution of care between men and women toward its collectivization [57]. Democracy itself is at risk if it is not protected [56]. This process involves not only redistributing tasks but also sharing doubts, knowledge, experiences, and emotional states, thereby reducing the isolation and loneliness in which care provision and reception often take place.

The results presented here allow for an understanding of care not as a domestic or merely affective practice, but as a structural category whose organization determines the possibilities for well-being, autonomy, and population health [4]. In the context of chronicity, this consideration is particularly relevant, as the prolonged and continuous care required to manage chronic diseases depends on who provides care, under what conditions, and with what institutional support [6].

The persistent invisibilization of care has direct repercussions on the social distribution of responsibilities. From the traditional separation between production and reproduction, between public and private, care has been relegated to the private sphere, assigning it mostly to women, stripping it of economic, social, and health-related recognition [7,58,59]. This structure has a particularly strong impact on chronic care, where caregiving demands are more prolonged and the need for supervision, accompaniment, and management increases as the disease progresses [8]. Thus, chronicity reproduces and amplifies patterns of inequality, as women—particularly mothers, daughters, and spouses—assume most of this indispensable yet unpaid and socially devalued work [9,60].

Regarding the dimensions of care, the findings confirm that chronicity requires the simultaneous consideration of material, subjective, and political dimensions [10]. The material–physical dimension becomes especially intensive in these contexts, as everyday tasks are transformed into complex, continuous, and often exhausting activities [16]. Insufficient public services and limited economic resources constrain families’ ability to outsource these tasks, creating a cycle of physical overload that negatively affects caregivers’ health. Notably, gender differences emerge in how this process is managed: while women tend to internalize the mandate to care to the point of exhaustion, men are more likely to externalize basic tasks, even at the cost of greater economic impoverishment [61,62]. This highlights how gender roles also shape responses to chronicity.

The subjective–relational dimension acquires particular relevance in chronic care, where emotional and relational needs intertwine with the progressive deterioration of the dependent person [61,63]. The caregiving experience becomes the axis of everyday life, generating both emotional gratification and high levels of stress, anxiety, guilt, social isolation, and compassion fatigue [61,64,65]. Although the emotional and affective dimension of care is fundamental, as pointed out by Mª Luz Esteban [9], it is important to avoid overemphasizing it, as doing so risks reinforcing the association between care and “the feminine.” Without denying the importance of emotions in caring for others, framing women as inherently more emotional—and therefore more capable of caring—is a social construction and one of the main instruments of social subordination, as it differentiates and hierarchizes contributions and spaces occupied by women and men.

The evidence suggests that the problem does not lie solely in caregiving activity itself, but rather in the conditions under which it is performed: lack of formal support, absence of respite services, rigidity of care provision, and strong moral pressure associated with the feminization of care [15]. This confirms that caregiver burden is not inherent to care, but to the social framework that regulates it [66]. Applying care ethics requires attention to particularism and context, as well as to the need to develop appropriate solutions in light of the available resources and the specific challenges faced [56].

From a political perspective, chronic care emerges as a space where structural inequalities and power relations converge [67]. The lack of institutional recognition perpetuates the notion that care is a private and feminine responsibility, thereby obscuring its contribution to sustaining public health [68]. The labor precarization of care workers, together with the absence of comprehensive policies guaranteeing rights, demonstrates that the organization of care directly reflects the political priorities of welfare systems [2,69].

Most notably, the findings show that chronic care is currently sustained through global care chains, in which migrant women play a fundamental role [70]. These workers perform essential tasks under highly vulnerable conditions, characterized by long working hours, limited labor regulation, low wages, and dependence on employers for legal or administrative matters [50]. The centrality of their work reveals, on the one hand, the inability of public systems to provide sufficient care, and on the other, the consolidation of a model that externalizes inequality onto women in precarious situations [52]. Global care chains thus function as an adjustment mechanism that allows chronic care to be sustained in countries with greater population ageing and higher socioeconomic levels, while simultaneously reproducing gender-, class-, and race-based inequalities [2].

Overall, this discussion highlights that the current model of chronic care is unsustainable and deeply inequitable. The combination of familistic models, reliance on precarious migrant labor, and the lack of robust public policies creates a scenario in which the burden of care is unevenly distributed, penalizing those who provide care—primarily women—and those who receive it—especially dependent older adults with limited resources. Therefore, it is essential to advance toward policies of social co-responsibility, not only by promoting caring masculinities [62], but also by strengthening public services [52], professionalizing the care sector, and formally recognizing care work. Only in this way will it be possible to guarantee a chronic care model that is sustainable, just, and respectful of the dignity of all those involved.

The main limitation of this study is that narrative reviews do not include a systematic assessment of the methodological quality of the included studies, which may introduce selection bias. However, this approach is appropriate for the aim of the study, which focuses on the conceptual, political, and structural analysis of care rather than on the quantitative synthesis of results [71].

Furthermore, as this study focuses on family care, the analysis of formal professional care provided within healthcare services remains beyond its scope. Relevant contributions from the clinical field, including concrete examples of chronic diseases (e.g., cancer, diabetes, cardiovascular diseases), were therefore excluded from the selection, although they would complement the reflections presented here.

As future proposals, it would be interesting to conduct research that delves deeper into:

(a) promote the production of information that enables informed diagnoses of the current organization of care and makes visible the contribution of unpaid work to economic functioning [6];
(b) contribute to building social demand in favor of public care policies that enable redistribution [28];
(c) develop integrated public policy proposals that expand individuals’ capacity to choose how care is organized and facilitate work–family reconciliation, exploring alternative forms of community living and other possibilities, including resistance to the gender mandate to care [9,21];
(d) help transform gender stereotypes surrounding care by denaturalizing its feminization [31,40];

In the healthcare field, these reflections lead to the following recommendations for health professionals:

(e) to examine approaches to care from a gender perspective within healthcare systems [20,29];
(f) to consider caregivers as subjects of care in their own right, rather than as objects or instruments supporting the care of people with chronic illness, incorporating a gender perspective [29,39];
(g) to actively involve men in family-based chronic care [29,32];
(h) to develop health education strategies for family caregivers and migrant paid caregivers, adopting an approach that addresses their triple vulnerability related to gender, ethnicity, and social class, without reinforcing or deepening such vulnerabilities [35,36].

## 5. Conclusions

Care is a structural, political, and transnational category that sustains life and healthcare systems. In the field of chronic care, its recognition, redistribution, and socialization are essential for social justice and for ensuring the dignity of both those who provide care—predominantly women—and those who receive it. Future research should empirically apply this framework to different welfare regimes and care systems in order to further assess its analytical and policy relevance.

## Data Availability

No new data were created or analyzed in this study.
